# Electricity transmission, distribution losses and economic growth in South Africa

**DOI:** 10.1016/j.heliyon.2020.e05564

**Published:** 2020-11-24

**Authors:** Samuel Adams, Francis Atsu, Edem Mensah Klobodu, Lamptey Richmond

**Affiliations:** a Ghana Institute of Management and Public Administration (GIMPA), School of Public Service and Governance, AH 50, Achimota, Accra-Ghana; b GIMPA Business School, AH 50, Achimota, Accra-Ghana; c Ghana Business School, AH 50, Achimota, Accra-Ghana

**Keywords:** Economics, Electrical engineering, Energy, Energy economics, Energy conservation, Energy policy, Electricity infrastructure, Transmission and distribution losses, Economic growth, South Africa, Autoregressive distributed lag

## Abstract

This paper employed the autoregressive distributed lag (ARDL) methodology to examine the effect of electricity transmission and distribution losses (ETL) on the economic growth of South Africa over the period 1971–2014. After controlling for foreign direct investment (FDI) and financial development, the results of the study show long-run negative relationship between ETL and economic growth. For robustness checks, we account for non-linearities/asymmetries in our model and find that a percentage increase in ETL decreases economic growth from 3.786% to 2.245%. The correction of the distortions of the convergence to long-run equilibrium by temporary shocks is reduced from 30.4% to 25.1%. Additionally, financial development and gross fixed capital formation promote growth while FDI and trade have insignificant effect.

## 1 Introduction

Energy infrastructure is a vital component of public infrastructure that facilitates economic growth. The efficiency effects of energy infrastructure are determined within the context of technology type, age of equipment, extent of periodic investment, corruption or poor governance, and type of maintenance employed (Best and Burke, 2018; Gaur and Gupta, 2016; Oyinlola et al., 2020). McKinsey (2015) indicates that promoting the development of power or energy sector has the potential to transform African economies. Accordingly, Ebhota and Tabakov (2018) describe the inadequacy of electricity supply as a major constraint on the economic growth of Africa, while Shokoya and Raji (2019), Trotter (2019) and Onat (2010) claim that it is an obstacle to Africa's growth.

**Figure 1:**
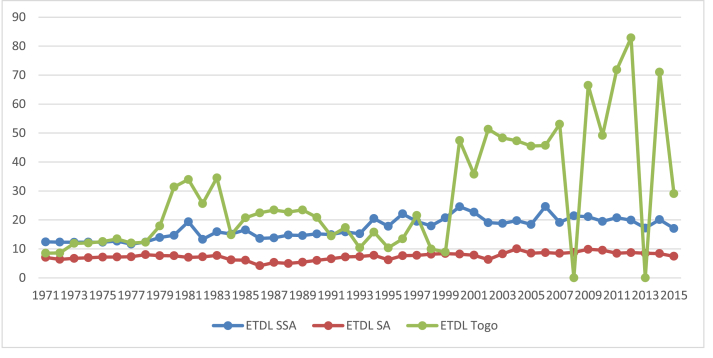
ETDL 1971–2015.

A key factor that needs attention in many African countries is the network and regulatory issues related to the adequacy of power supply and the efficiency in the transmission and distribution of electricity. Obviously, with the rich energy resources on the continent, there is no reason why the region is still beset with energy poverty challenges (Market Intelligence Report, 2018). The Frost and Sullivan (2015) report, for example, indicates that the high ETL losses (about 18%) in sub-Sahara Africa (SSA) impacts negatively on the continent's growth prospects. The South African ETL, however, compares with world average of 10%. Between 1971 and 1997, it was only in 1978 that the ETL went beyond 8% and over the same period, the least loss occurred in 1986 with a value of 4. 20%. There has been an increasing trend in the ETL since 1998 with an average loss of 8.50 % and the highest loss occurring in 2004 at 10%, compared to the highest for the SSA region at 24% in 2000 and Togo's of 83% in 2012 as seen in Figure 1 (World Bank, 2019).

**Figure 2:**
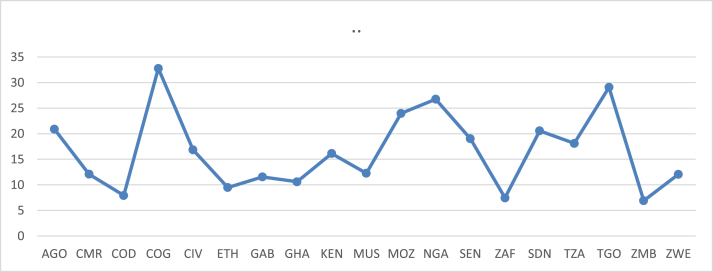
Average Electricity distribution and transmission losses.

On the average, however, the average ETL for Togo is about 29%, which is exceeded only by that of Congo at 33% over the period 1971–2015 (See Figure 2).

In this study, electric power transmission and distribution losses are defined as deviations between power that is generated and actually distributed to consumers, due to both technical and nontechnical factors (World Bank, 2015; Klynveld Peat Marwick Goerdeler [KPMG], 2015; Sadoyskaia et al., 2019; Shokoya and Raji, 2019). The losses amount to about US$ 5 billion annually in SSA with South Africa alone contributing about US$ 1.5 billion (Ecobank, 2014; Shokoya and Raji, 2019). Additionally, the ETL has an impact on electricity demand and environmental degradation (Costa-Compi et al., 2018a; Costa-Campi et al., 2018b; Surana and Jordana, 2019). Even under the umbrella of smart grids, the losses are still growing which suggests that efficient and reliable generation of electricity but more critically its transmission is linked to the very survival of human livelihoods (Kumar et al., 2017).

Recent research suggests that ETL has an effect on economic growth because most of these losses are wasted resources, however not much empirical studies have been conducted on the ETL– growth nexus. This gap motivates the study. It is worth mentioning though that many studies have considered the energy usage and economic performance relationship (Zheng and Walsh 2019; Adams et al., 2018; Churchill and Ivanovski, 2020; Humbatova et al., 2020; Costa-Campi et al., 2018a). The study is relevant to the extant literature in three ways. Foremost, the findings of the study will provide information to help to not only quantify but also monitor and more importantly manage the problems associated with ETL. Second, we identify asymmetric effects or nonlinearities (using the NARDL), if any, to determine the differential effects of changes of ETL on economic output. Third, we account for trade, FDI, and financial development to test how the integration into the world economy affects ETL– economic growth relationship. In studying the South African case, it is important to note that it is exceptionally energy intensive but also more efficient compared to most SSA countries. Most of South Africa energy (about 95%) is supplied by Eskom and the other 5% by Independent Power producers [IPP](ESKOM, 2015a; Mokveld and von Eije, 2018; Ratshomo and Nembahe, 2018). South Africa is the only SSA country with a functional nuclear energy plant (Koeberg) for electricity generation of about 2000 MW, which is expected to increase to 9600 MW by 2030. South Africa is noted for its rich reserves of uranium for nuclear reactors estimated at nearly 280, 000t, which is about 5% of the global reserves. Despite the huge electricity generation capacity in South Africa, the country experienced a power crisis in 2008 and marginally in 2014/2015. These were due in part to policy and administrative failures and regulatory uncertainty mostly related to the performance of ESKOM which was indebted to the tune of nearly US$30 billion.

In the past two decades, South Africa has diversified its sources of energy from coal towards renewable energy (Department of Energy, 2015), and it is known to be more successful than other countries on the continent in deploying renewables (Numbi et al., 2014; ESKOM, 2015a, ESKOM, 2015b; BP Statistical Review of World Energy, 2018). In addition, the South African government plans to introduce a carbon tax on greenhouse gas emissions (Alton et al., 2014), while the cost of production of renewables is reducing around the world (Spalding-Fecher et al., 2017; World Energy Outlook, 2018; BP Statistical Energy Review, 2019). More important, South Africa is ranked number one emitter of Greenhouse gases in Africa and 14^th^ globally (Mokveld and von Eije, 2018). The unique position of South Africa as one of the richest SSA countries, the most energy intensive, most efficient in SSA and yet with ETL losses of over US$ I billion makes it worth studying to provide lessons to other countries in the region.

We employ the autoregressive distributed lag (ARDL) and non-linear ARDL (NARDL) methodologies which examine long-run relationship and account for asymmetries between the variables of interest over the period 1971–2014. In the sections that follow, the methodology is clearly defined, after which the analyses of results are presented, conclusions given and policy implications offered.

## 2 Data and model specification

We proceed in this section as follows. Section 2.1 captures the dataset for the analysis spanning the period 1971–2014. Section 2.2 offers the specification of the linear ARDL model, and Section 2.3 presents the asymmetric or nonlinear ARDL technique (NARDL) for robustness checks.

### 2.1 Data

The dependent variable used in this study is GDP per capita and ETL as the main independent variable. Further, we control for the effects of (i) trade openness, (ii) gross fixed capital formation, (iii) FDI and (iv) financial development. Refer to Table 1 for more details.

**Table 1:** Variable definition and sources.

No	Variable	Definition	Sources
1	*gdp*	Real Gross domestic product per capita	WDI
2	*bmoney*	Broad money (% of GDP)	WDI
3	*dcfs*	Domestic credit provided by financial sector (% of GDP)	WDI
4	*dcps*	Domestic credit to private sector (% of GDP)	WDI
5	*dcbs*	Domestic credit to private sector by banks (% of GDP)	WDI
6	*fdi*	Foreign direct investment, net inflows (% of GDP)	WDI
7	*gfcf*	Gross fixed capital formation (% of GDP)	WDI
8	*trade*	Trade (% of GDP)	WDI
9	*etl*	Electric power transmission and distribution losses (% of output)	WDI

Notes: WDI denotes the world development indicators database of the World Bank.

### 2.2 Model specification

The study employs a simple production model where we input etl as an additional factor of production (see equation 1) (Hulten, 1996; Straub et al., 2008).

(1) $$lgdp_{t}=\beta_{1}+\beta_{2}letl_{t}+\beta_{3}ltrade_{t}+\beta_{4}lfin_{t}+\beta_{5}lfcf_{t}+\beta_{5}lfdi_{t}+\varepsilon_{t}$$

where the gross domestic product per capita is represented by *gdp*, *etl* is electric power transmission and distribution losses share in GDP, *gfcf* is gross capital formation share in GDP, *trade* is trade openness as a percentage of GDP, *fin* is financial development, and *fdi is* foreign direct investment. The terms$\beta_{0},\cdots ,\beta_{6}$ represent the coefficients of the variables to be estimated, *l* denotes natural logarithm operator and $\varepsilon_{t}$ is the error.

#### 2.2.1 Symmetric ARDL model specification

The inability of the ordinary least squares (OLS) method in directly estimating nonstationary time series calls for more sophisticated models. Of particular importance to this study is Pesaran et al.’s (2001) ARDL method which is suitable for modelling mixed order of integration less than 2. Additionally, this technique can be set-up in a single equation and variables modelled with uneven lag-lengths. The first stage of ARDL “bounds test” involves estimating long-run associations of the variables and the coefficients are then estimated if cointegration is established. The F-statistic is used for Bounds testing for the presence of a long-run relationship. To achieve the research objective, we adopt the standard ARDL (p, q) model and specify our model as follows:

(2) $$\text{Δ}lgdp_{t}=\alpha_{0}+\gamma_{1}lgdp_{t-1}+\gamma_{2}letl_{t-1}+\gamma_{3}ltrade_{t-1}+\gamma_{4}lfin_{t-1}+\gamma_{5}lgfcf_{t-1}+\gamma_{6}lfdi+\sum_{i=1}^{p}\beta_{1i}\text{Δ}lgdp_{t-i}+\sum_{i=0}^{q}\beta_{2i}\text{Δ}letl_{t-i}+\sum_{i=0}^{q}\beta_{3i}\text{Δ}ltrade_{t-i}+\sum_{i=0}^{q}\beta_{4i}\text{Δ}lfin_{t-i}+\sum_{i=0}^{q}\beta_{5i}\text{Δ}lgfcf_{t-i}+\sum_{i=0}^{q}\beta_{6i}\text{Δ}lfdi_{t-1}+\varepsilon_{t}$$

whereΔdenotes the first difference operator,$\alpha_{0}$ is a constant, $\gamma_{1},\cdots ,\gamma_{6}$ are coefficients on the lagged levels of the dependent and independent variables, $\beta_{1},\cdots ,\beta_{6}$ are the coefficients of the differenced lagged dependent and independent variables with the corresponding *p* and *q* optimal lag lengths, and $\varepsilon_{t}$represents the error term. In Eq. (2) the long-run or cointegration can be tested as follows: null hypothesis of no cointegration ($H_{0}:\gamma_{1}=\gamma_{2}=\gamma_{3}=\gamma_{4}=\gamma_{5}=\gamma_{6}=0$) as against an alternate hypothesis of existence of cointegration ($H_{a}:\gamma_{1}\neq \gamma_{2}\neq \gamma_{3}\neq \gamma_{4}\neq \gamma_{5}\neq \gamma_{6}\neq 0$) through an F test statistic. An F-statistic greater than the upper bound value indicates significant long-run relationship suggesting we fail to accept the null hypothesis of non-existence of cointegration otherwise we fail to reject the null hypothesis. Further, if the F statistic falls within the lower and upper bounds the implication would be that the test is inconclusive. We use the Akaike Information Criterion (AIC) since it deals with the trade-off between complexity and goodness-of-fit to determine the order of the ARDL model. The next step involves estimating the ECM along with the short-run parameters. As expected, the sign of the error correction (coefficient of the lagged error correction term-ECT) must be negative and statistically significant to confirm that short-run dynamics eventually converges to long-run equilibrium.

We investigate the stationarity of the variables based on the Augmented Dickey-Fuller (ADF) and Phillips-Perron (PP) tests. Additionally, we use the Zivot-Andrews (ZA) test to check whether structural breaks affect our unit root tests. Specifically, the null hypothesis of ZA assumes time series has a unit root with structural breaks.

#### 2.2.2 Asymmetric or nonlinear ARDL (NARDL) model specification

Cointegration tests assume that the cointegrating vector is symmetric during the entire study period, however, there is a possibility that short-run or long-run relationship is nonlinear in nature. For robustness checks on our model, following the works of Shin et al. (2011), we modify our ARDL estimator to a nonlinear ARDL model by incorporating short- and long-run nonlinearities through positive and negative partial sum decompositions of the independent variables represented in Eqs. (3) and (4):

(3) $$X_{t}^{+}=\sum_{i=1}^{t}\text{Δ}X_{i}^{+}=\sum_{i=1}^{t}\max\left(\text{Δ}X_{i},0\right)$$

(4) $$X_{t}^{-}=\sum_{i=1}^{t}\text{Δ}X_{i}^{-}=\sum_{i=1}^{t}\min\left(\text{Δ}X_{i},0\right)$$

From Eqs. (3) and (4), we obtain the NARDL framework that decomposes the independent variables into the partial sums of their positive and negative changes, parameterized by

(5) $$\text{Δ}lgdp_{t}=\delta +\rho lgdp_{t-1}+\theta_{1}^{+}letl_{t-1}+\theta_{1}^{-}letl_{t-1}+\theta_{2}^{+}ltrade_{t-1}+\theta_{2}^{-}ltrade_{t-1}+\theta_{3}^{+}lfin_{t-1}+\theta_{3}^{-}lfin_{t-1}+\theta_{4}^{+}lgfcf_{t-1}+\theta_{4}^{-}lgfcf_{t-1}+\theta_{5}^{+}lfdi_{t-1}+\theta_{5}^{-}lfdi_{t-1}+\sum_{i=1}^{p}\alpha_{i}\text{Δ}lgdp_{t-1}+\sum_{i=0}^{q+}\omega_{1i}^{+}\text{Δ}lelt_{t-1}+\sum_{i=0}^{q-}\omega_{1i}^{-}\text{Δ}lelt_{t-1}+\sum_{i=0}^{q+}\omega_{2i}^{+}\text{Δ}ltrade_{t-1}+\sum_{i=0}^{q-}\omega_{2i}^{-}\text{Δ}ltrade_{t-1}+\sum_{i=0}^{q+}\omega_{3i}^{+}\text{Δ}lfin_{t-1}+\sum_{i=0}^{q-}\omega_{3i}^{-}\text{Δ}lfin_{t-1}+\sum_{i=0}^{q+}\omega_{4i}^{+}\text{Δ}lgfcg_{t-1}+\sum_{i=0}^{q-}\omega_{4i}^{-}\text{Δ}lgfcf_{t-1}+\sum_{i=0}^{q+}\omega_{5i}^{+}\text{Δ}lfdi_{t-1}+\sum_{i=0}^{q-}\omega_{5i}^{-}\text{Δ}lfdi_{t-1}$$

where $\theta_{i}^{+}$’s and $\theta_{i}^{-}$’s are the asymmetric positive and negative effects of the independent variables on the dependent variable with the respective q+and q− optimal lag lengths. In the fashion of Shin et al. (2011), the positive and negative long-run coefficients are computed from Eq. (5) as $L_{X^{+}}=\theta_{X}^{+}/\rho$and$L_{X^{-}}=\theta_{X}^{-}/\rho$, respectively. Afterwards, we validate our cointegration or long-run relationship by setting the coefficients of the lagged levels of the variables to zero. This is achieved by using the F-statistic, which tests the null hypothesis of no asymmetric cointegration.

($H_{0}:\theta_{1}=\theta_{2}=...=\theta_{6}$) as against an alternative of existence of asymmetric cointegration relationship ($H_{a}:\theta_{1}\neq \theta_{2}\neq ...\neq \theta_{6}$). Existence of long run asymmetry effects (the coefficients) is implied by rejecting the following null of$\theta_{i}^{+}=\theta_{i}^{-}$.

## 3 Empirical analysis

This section is organized as follows: Section 3. 1 presents the summary statistics of the variables, including descriptive statistics, pairwise correlation matrix and test scale for the composite measure of financial development. Section 3.2 offers unit root tests using ADF, PP, and ZA tests. Section 3.3 captures the regression output and discussions of ARDL and NARDL models for robustness checks.

### 3.1 Summary statistics

The descriptive statistics and pairwise correlations are presented in Panel I and Panel II of Table 2, respectively. Real GDP per capita and *letl* have averages of 8.017 and 1.994 over the years (1971–2014), while the averages of the remaining variable are within the range -0.174 and 4.783. The values of *lfdi* and *ltrade* are the most and least dispersed variables, respectively. The regressors *letl, lgdp, ltrade, lfdi*, and *ldcps* are negatively skewed, while the rest are positively skewed. Except *letl* and *lfdi*, all the variables exhibit negative excess kurtosis.

**Table 2:** Descriptive statistics and pairwise correlation.

Panel 1: Descriptive statistics										
Statistic	*lgdp*	*letl*	*ltrade*	*lfdi*	*lgfcf*	*lfin*	*ldcbs*	*ldcfs*	*ldcps*	*lbmoney*
Mean	8.017	1.994	3.943	-0.714	3.056	4.349	4.038	4.783	4.544	4.063
Median	8.025	2.034	3.942	-0.592	3.021	4.318	4.011	4.831	4.654	4.034
Max.	8.988	2.303	4.289	1.789	3.469	4.767	4.360	5.261	5.076	4.392
Min.	6.799	1.434	3.624	-5.993	2.718	3.977	3.726	4.306	3.988	3.818
Std. Dev.	0.551	0.180	0.149	1.653	0.227	0.246	0.176	0.331	0.368	0.151
Skewness	-0.200	-0.890	-0.160	-1.283	0.228	0.255	0.237	0.107	-0.025	0.744
Kurtosis	2.581	3.970	2.714	4.884	1.748	1.580	1.792	1.350	1.361	2.524
JB	0.613	7.539	0.338	14.784	3.255	4.174	3.016	4.957	4.816	4.476
Prob.	0.736	0.023	0.845	0.001	0.196	0.124	0.221	0.084	0.090	0.107
N	44	44	44	44	44	44	44	44	44	44
Panel II: Pairwise correlations										

	*lgdp*	*letl*	*ltrade*	*lfdi*	*lgfcf*	*lfin*	*ldcbs*	*ldcfs*	*ldcps*	*lbmoney*

*lgdp*	*1.000*									
*letl*	0.649	1.000								
*ltrade*	0.416	0.367	1.000							
*lfdi*	0.239	0.301	0.600	1.000						
*lgfcf*	-0.510	-0.296	0.164	-0.123	1.000					
*lFin*	0.823	0.727	0.511	0.499	-0.622	1.000				
*ldcbs*	0.772	0.711	0.484	0.520	-0.586	0.969	1.000			
*ldcfs*	0.819	0.701	0.425	0.452	-0.724	0.985	0.937	1.000		
*ldcps*	0.827	0.685	0.360	0.419	-0.749	0.977	0.945	0.989	1.000	
*lbmoney*	0.525	0.582	0.818	0.532	0.083	0.688	0.646	0.583	0.525	1.000

*Notes*: *Notes*: *lfin* is the composite measure of financial development computed from the individual measures of financial development (*ldcps, ldcbs, ldcfs*, and *lbmoney*) as shown in Table 3.

The pairwise correlation matrix in Panel II of Table 2 shows that the financial development variables (domestic credit to private sector, domestic credit to private sector provided by banks, domestic credit to private sector provided by financial sector, and broad money) and the composite measure are highly correlated. The test scale has a reliability value of 0.907 which is more appropriate since it is far beyond acceptable range of 0.7–0.8. The other correlations are not very high and this does not pose any problem in the parameter estimations. In Table 3, following Law et al. (2018), we conduct item-analysis for the financial development composite scale using the Cronbach's Alpha approach.

**Table 3:** Item-analysis for financial development.

Item	Obs	Sign	Item-test correlation	Item-rest correlations	Alpha
*ldcps*	44	+	0.982	0.953	0.840
*ldcbs*	44	+	0.975	0.963	0.867
*ldcfs*	44	+	0.989	0.974	0.810
*lbmoney*	44	+	0.695	0.601	0.949
*Test scale (fin)*					0.907

Notes: We calculate the Cronbach's Alpha using the STATA “alpha” where values within the range 0.700 and above are more appropriate.

### 3.2 ADF, PP and ZA unit root tests

We employ the ADF and PP unit root tests to assess the stationarity of the time series data. Panel I and Panel II present the unit root tests in levels and first difference respectively (See Table 4). Most of the variables except FDI become stationary only after first difference suggesting that the variables are integrated of order one: I (1). After assessing the unit roots of all the series using the two tests, the study goes further to check whether variables remain a combination of I (0) and I (1) series if structural breaks are encountered. This is done by using the Zivot-Andrews (ZA) test and the results are presented in Table 5.

**Table 4:** Unit root tests.

Variable	ADF-Test		PP-test		Outcome
	Const	Const + Trend	Const	Const + Trend	
**Panel I: Levels**					
*lgdp*	-1.832	-3.092	-1.818	-2.755	
*letl*	-2.254	-2.860	-2.254	-2.773	I (1)
*ltrade*	-1.613	-1.804	-1.569	-1.762	I (1)
*lfin*	-0.287	-3.162	-0.417	-3.162	I (1)
*lgfcf*	-1.725	-1.808	-1.371	-1.374	I (1)
*lfdi*	-3.298∗∗	-3.902∗∗	-3.209∗∗	-3.713∗∗	I (0)/I (1)
**Panel II: First differenced**					
*lgdp*	-5.397∗∗∗	-4.199∗∗∗	-4.356∗∗∗	-4.490∗∗∗	I (1)
*letl*	-8.496∗∗∗	-8.385∗∗∗	-8.734∗∗∗	-8.615∗∗∗	I (1)
*ltrade*	-5.938∗∗∗	-5.883∗∗∗	-6.087∗∗∗	-6.413∗∗∗	I (1)
*lfin*	-9.287∗∗∗	-9.247∗∗∗	-9.491∗∗∗	-9.690∗∗∗	I (1)
*lgfcf*	-4.421∗∗∗	-4.439∗∗∗	-9.491∗∗∗	-4.133∗∗∗	I (1)
*lfdi*	-7.791∗∗∗	-7.681∗∗∗	-8.335∗∗∗	-8.170∗∗∗	I (0)/I (1)

Note: ∗∗∗/∗∗ denote significance at 1% and 5% levels, respectively.

**Table 5:** Zivot Andrews Unit root test.

Variable	Levels		Break date	Difference		Outcome
	t-Statistic	Probability		t-Statistic	Probability	
*lgdp*	-3.640∗∗	0.020	1996	-5.250	0.007	I (1)
*ltrade*	-3.260	0.204	1982	-6.113	0.003	I (0)/I (1)
*lgfcf*	-3.686∗∗∗	0.007	2005			I (0)
*letl*	-4.688∗	0.001	1984	-9.323	0.010	I (0)/I (1)
*lfdi*	-3.574	0.131	2000	-6.118	0.002	I (1)
*lfin*	-4.353∗∗∗	0.001	1992			I (0)

Note: ∗∗∗, ∗∗, ∗ denote significance at 1%, 5% and 10% levels, respectively. The break dates are endogenously determined.

The results in Table 5 confirm that each variable is either I (0) or I (1) for which we can proceed with the ARDL model estimations.

### 3.3 Regression output discussions

The F-statistic in Panel I of Table 6a shows a robust long-run relationship between the variables based on ARDL specification ARDL (2, 2, 1, 1, 2, 1). We perform the diagnostic testing of our model as follows: (i) serial correlation is examined using Breusch-Pagan-Godfrey LM serial correlation with the null of no serial correlation, (ii) heteroscedasticity is assessed using ARCH and Breusch-Godfrey tests with the nulls of homoscedasticity, and (iii) specification errors (e.g. omitted variables, incorrect functional form) are investigated using Ramsey RESET test with the null of correct specification. The results presented in Panel II of Table 6a show that the projected relationships passed the diagnostic tests as we are unable to reject the null of each one of the tests at 1% significance level.

**Table 6a:** Test of Long-run relationship.

Panel I: Bounds test				
ARDL specification	F-statistic	SL	I (0)	I (1)
ARDL (2, 2, 1, 1, 2, 1)	5.433∗∗∗	10%	2.080	3.000
		5%	2.390	3.380
		1%	3.060	4.150
Panel II: Residual Diagnostics and function form	statistic	p-value		
ARCH heteroscedasticity	0.168	0.686		
Breusch-Pagan-Godfrey heteroscedasticity	1.726	0.153		
Breusch-Pagan-Godfrey LM serial corr.	3.628	0.080		
Ramsey RESET specification error.	1.735	0.105		

Notes: ∗∗∗denotes significance at 1% level.

Given the existence of long-run equilibrium association, we estimate the short-run and long-run regression coefficients which are respectively presented in Panel I and Panel II of Table 6b below. The findings show that ETL has a significant negative effect on output growth. Specifically, 1% percent increase in etl reduces economic growth by 0.561% and 3.786% in the short-run and long run, respectively. Financial development is positive and significant such that a 1% increase of same promotes economic growth in the short-run by 0.941% and in the long-run by 5.368%.

**Table 6b:** ARDL parameter estimates.

Panel I: Short-run coefficients			
Variable	Coefficient	Std. Error	Prob.
*d (lgdp (-1))*	0.485∗∗∗	0.123	0.001
*d (ltrade)*	-1.662∗∗∗	0.256	0.000
*d (ltrade (-1))*	-0.972∗∗∗	0.219	0.001
*d (lfdi)*	-0.002	0.008	0.823
*d (lfin)*	0.941∗∗∗	0.232	0.001
*d (lgfcf)*	1.261∗∗∗	0.301	0.001
*d (lgfcf (-1))*	-0.817∗∗∗	0.228	0.003
*d (letl)*	-0.561∗∗∗	0.181	0.007
*ect (-1)*	-0.304∗∗∗	0.042	0.000
Panel II: Long-run coefficients			
Variable	Coefficient	Std. Error	Prob.
*ltrade*	-1.424∗	0.801	0.096
*lfdi*	-0.063	0.053	0.256
*lfin*	5.368∗∗∗	1.040	0.000
*lgfcf*	1.146∗∗	0.437	0.019
*letl*	-3.786∗∗	1.507	0.024
C	-5.598	1.713	0.005

Note: ∗∗∗, ∗∗, ∗ denote significance at 1%, 5% and 10% levels, respectively.

Similarly, a percentage increase in gross fixed capital formation (gfcf) leads to an increase in economic growth by an increase in economic growth by 1.261% and 1.146% in the short and the long run respectively. Trade is statistically significant in the short-run but insignificant in the long-run, while foreign direct investment (fdi) is statistically insignificant.

**Figure 3:**
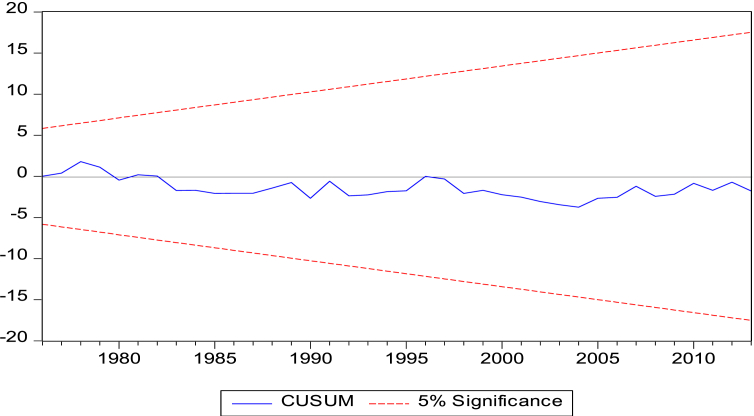
Cumulative Sum of Recursive Residuals Graph. Note: Lines depict critical bounds at 5% level of Significance.

**Figure 4:**
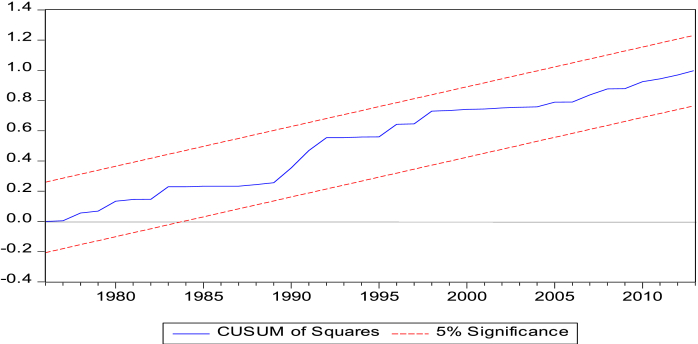
Cumulative Sum of Squares Recursive Residuals Graph. Note: Lines depict critical bounds at 5% level of Significance.

The ect coefficient (-0.304) in Panel I of Table 6b suggests that deviations from equilibrium are corrected at a rate of 30.4% per year. Further, we assess the stability of our estimates (short-run and long-run) over the study period (1971–2014). We are informed by the cumulative sum of recursive residuals (CUSUM) and cumulative sum of squares of recursive residuals (CUSUMSQ) tests that our models are stable over the study period. Pictorially the CUSUM and CUSUMSQ show that our plots lie within the critical bounds of 5% significance indicating stability of our parameters (see Figures 3 and 4).

For robustness checks, we investigate the asymmetries using NARDL technique. Prior to estimating the coefficients of NARDL model, we conduct the bounds test to assess the long-run nonlinear relationship. The F-statistic (3.908) presented in Table 7 exceeds the upper bound of our critical value at 1% level of significance, thus we conclude there are asymmetric cointegration or relationship between our variables. The long-run coefficients are presented in Table 8.

**Table 7:** Bounds test for nonlinear cointegration.

Panel I: Bounds test				
NARDL specification	F-statistic	Sign. Level	I (0)	I (1)
NARDL (1, 0, 2, 1, 0, 2, 0, 2, 0, 1, 1)	3.908	10%	1.760	2.770
		5%	1.980	3.040
		1%	2.410	3.610
Panel II: Residual Diagnostics	Statistic	p-value		
ARCH heteroscedasticity	0.014	0.908		
Breusch-Pagan-Godfrey heteroscedasticity	0.570	0.891		
Breusch-Pagan-Godfrey LM serial	6.500	0.020		
Ramsey RESET specification error.	0.033	0.974		

Notes: The model passed all the diagnostic tests at 1% significant level.

**Table 8:** Nonlinear ARDL estimation results.

Variable	Coefficient	Std. Error	Prob.
*ltrade_pos*	1.319	2.818	0.645
*ltrade_neg*	3.565∗∗	1.694	0.048
*lfdi_pos*	-0.592∗∗	0.273	0.043
*lfdi_neg*	-0.242	0.176	0.184
*lfin_pos*	3.957	2.697	0.158
*lfin_neg*	0.696	1.566	0.662
*lgfcf_pos*	-0.304	3.264	0.927
*lgfcf_neg*	0.693	1.152	0.554
*letl_pos*	2.162∗	1.102	0.064
*letl_neg*	-2.245∗∗	0.881	0.019
C	7.522	0.677	0.000
***Long-run equilibrium relationship***		0.251∗∗∗	

Note: ∗∗∗, ∗∗, ∗ denote significance at 1%, 5% and 10% levels, respectively.

Concentrating on the main variable of interest, the results show that while an increase in the ETL reduces economic growth, a reduction in the ETL has the opposite effect. Specifically, a percentage decrease in electricity transmission and distribution losses causes economic growth to increase by 2.162%, which shows the existence of asymmetric effects. The long-run equilibrium relationship is corrected by 25.1% if the relationship experiences a temporary shock.

## 4 Conclusion and policy implications

The paper investigated the impact of ETL on output growth of South Africa spanning 44 years (1971–2014). Using the ARDL and non-linear ARDL to account for asymmetric effects. The results of the study show that ETL has a negative effect on output growth. Furthermore, financial development and gross fixed capital formation enhance growth in South Africa, while foreign direct investment does not contribute to output growth. The results are robust to structural shifts and breaks.

Four policy implications emerge from the analyses. Primarily, policy makers in South Africa should consider the medium to the long terms effects of ETL on output growth. Investments in new production plants, inventories and other assets by investors is correlated with availability and reliability of energy. From a signalling effect perspective, captains of industry and investors lose confidence in an economy experiencing high ETL with negative repercussions on economic growth (Brem et al., 2020). Policy makers should be encouraged to be innovative and implement energy sector policies that consider both short and long-term implications.

Second, there is the need for policymakers to prioritize investment in the energy sector considering population growth and the expansion of the rural economy. In this sense, investments should be directed towards upgrade of existing infrastructures including transmission and distribution lines. With respect to energy production, policies aimed at attracting investments through appropriate mix of state power producers and independent power producers can be highly beneficial. Further, in developing such policies an appropriate balance between ESKOM and IPPs in view of power asymmetries require thoughtful consideration. In this light the positive effect financial development is worth emphasizing as it is consistent with the Schumpeterian thesis and Levine's argument of finance being critical to innovation and productivity that are key ingredients of economic growth. However, the insignificant effect of FDI is worth studying, and we suggest future studies should look at the issue comprehensively either through the complementarity or threshold effects to provide more evidence informed policy for South Africa, and possibly the SSA region at large (Yeboua, 2019).

Third, diversification of energy production sources such as renewable energy will immensely benefit the entire nation (Sarkodie and Adams, 2020; Cook and Elliot, 2020). As the various International Renewable Energy Agency, 2013, International Trade Administration, 2017, 2018) and Market Intelligence Report (2018) show, renewable energy consumption have the twin benefits of reduction in environmental degradation and investment in equipment and machinery (Adams and Acheampong, 2019). With the declining cost in the technology and deployment of renewable energy, the diversification option becomes all the more pertinent. Obviously, as the continent with highest rural population and energy poverty, the vast renewable resources make its development and use imperative. Even as the use of renewables grows in the SSA region, it is important that this is encouraged even further to ensure energy security.

Finally, legal considerations must be embedded in policy development and implementation to prevent delays arising from legislative barriers, policy uncertainty, and institutional failures that inhibit innovative capability and financial sector development needed for the power sector (Brown, 2019; Adams and Klobodu, 2017). The issues raised above suggest both bureaucratic capacity and regulatory effectiveness will be critical in pursuing forward looking and dynamic energy sector to meet the aspirations of the South African people. In conclusion, South Africa's energy situation and events provide important lessons to most SSA countries who are experiencing severe transmission and distribution losses. Failure to continuously invest resources in energy generation, transmission and distribution infrastructure could lead to economic crisis if nothing is done as energy inefficiencies have enormous effects on the financial sustainability of the power and utilities sectors in general. This is because reduction in ETL would lead to improvement in the revenue base, enhance investment and capitalization in the power sector to promote output growth and subsequently reduction in environmental pollution.

## Declarations

### Author contribution statement

Samuel Adams: Conceived and designed the experiments; Contributed reagents, materials, analysis tools or data; Wrote the paper.

Francis Atsu: Analyzed and interpreted the data; Contributed reagents, materials, analysis tools or data.

Edem Klobodu Mensah: Analyzed and interpreted the data; Wrote the paper.

Richmond Lamptey: Performed the experiments; Wrote the paper.

### Funding statement

This research did not receive any specific grant from funding agencies in the public, commercial, or not-for-profit sectors.

### Competing interest statement

The authors declare no conflict of interest.

### Additional information

No additional information is available for this paper.

## References

[bib1] Adams S., Acheampong A.O. (2019). Reducing carbon emissions: the role of renewable energy and democracy. J. Clean. Prod..

[bib4] Adams S., Klobodu E.K.M., Lamptey R.O. (2017). Electric power transmission, distribution losses, and economic growth in Ghana. Social, Health, and Environmental Infrastructures for Economic Growth.

[bib5] Adams S., Klobodu E.K.M., Apio A. (2018). Renewable and non-renewable energy, regime type and economic growth. Renew. Energy.

[bib12] Alton T., Arndt C., Davies R., Hartley F., Markrelov K., Thurlow J., Ubogu D. (2014). Introducing carbon taxes in South Africa. Appl. Energy.

[bib17] Best R., Burke P.J. (2018). Electricity availability: a precondition for faster economic growth?. Energy Econ..

[bib19] (2019). BP Statistical Review of World Energy 2019. London, UK.

[bib20] Brem A., Nylund P., Viardot E. (2020). The impact of the 2008 financial crisis on innovation: a dominant design perspective. J. Bus. Res..

[bib25] Churchill S.A., Ivanovski K. (2020). Electricity consumption and economic growth across Australian states and territories. Appl. Econ..

[bib26] Cook T., Elliott D. (2020). Renewable energy in Africa: changing support systems. Renewable Energy and Sustainable Buildings.

[bib27] Costa-Campi M.T., García-Quevedo J., Trujillo-Baute E. (2018). Electricity regulation and economic growth. Energy Pol..

[bib28] Costa-Campi M.T., Daví-Arderius D., Trujillo-Baute E. (2018). The economic impact of electricity losses. Energy Econ..

[bib29] Department of Energy (2015). South Africa’s energy situation. Energy advocacy.

[bib31] Ebhota W.S., Tabakov P.Y. (2018). Power inadequacy, the thorn in economic growth of Nigeria. Int. J. Appl. Eng. Res..

[bib32] Ecobank (2014). Middle Africa Insight Series, Power.

[bib35] ESKOM (2015). Company Information. http://www.eskom.co.za/OurCompany/CompanyInformation/Pages/Company_Information_1.aspx.

[bib36] ESKOM (2015). Tariff and Charges. http://www.eskom.co.za/CustomerCare/TariffsAndCharges/Pages/Tariffs_And_Charges.aspx.

[bib37] Frost & Sullivan report (2015). Electricity Distribution and Power Challenges Create Investment Opportunities for Africa. 15th Africa Utility Week Conference. Cape Town.

[bib38] Gaur V., Gupta E. (2016). The determinants of electricity theft: an empirical analysis of Indian states. Energy Pol..

[bib39] Hulten C.R. (1996). Infrastructure Capital and Economic Growth: How Well You Use it May Be More Important than How Much You Have (No. W5847).

[bib40] Humbatova S.I., Ahmadov F.S., Seyfullayev I.Z., Hajiyev N.G.O. (2020). The relationship between electricity consumption and economic growth: evidence from Azerbaijan. Int. J. Energy Econ. Pol..

[bib44] International Renewable Energy Agency (2013). IRENA Southern Africa Power Pool: Planning and Prospects for Renewable Energy.

[bib45] International Trade Administration (2017). South Africa-electrical Power Systems. https://www.export.gov/apex/article2?id=South-Africa-electrical-power.

[bib48] Klynveld Peat Marwick Goerdeler [KPMG] (2016). Sector Report: Power in Africa. https://www.kpmg.com/Africa.

[bib50] Kumar S., Pal G., Shah T. (2017). High performance overhead power lines with carbon nanostructures for transmission and distribution of electricity from renewable sources. J. Clean. Prod..

[bib51] Law S.H., Lee W.C., Singh N. (2018). Revisiting the finance-innovation nexus: Evidence from a non-linear approach. J. Innov. Knowl.

[bib52] Market Intelligence Report (2018). An African Energy Industry Report. Ispy Publishing’s Industry Survey, Market Intelligence, and Forecasts Series.

[bib53] McKinsey (2015). Brighter Africa: the growth potential of the sub-Saharan electricity sector. https://www.mckinsey.com/industries/electric-power-and-natural-gas/our-insights/powering-africa%20on%2014/01/2018.

[bib55] Mokveld K., von Eije S. (2018). South Africa Energy Sector Report.

[bib56] Numbi B.P., Zhang J., Xia X. (2014). Optimal energy management for a jaw crushing process in deep mines. Energy.

[bib57] Onat N. (2010). Transmission and distribution losses of Turkey's power system. WSEAS International Conference on Energy Planning, Energy Saving.

[bib58] Oyinlola M.A., Adedeji A.A., Bolarinwa M.O., Olabisi N. (2020). Governance, domestic resource mobilization, and inclusive growth in sub-Saharan Africa. Econ. Anal. Pol..

[bib62] Pesaran M.H., Shin Y., Smith R.J. (2001). Bounds testing approaches to the analysis of level relationships. J. Appl. Econom..

[bib64] Ratshomo K., Nembahe R. (2018). South African Energy Sector Report 2018.

[bib65] Sadovskaia K., Bogdanov D., Honkapuro S., Breyer C. (2019). Power transmission and distribution losses–A model based on available empirical data and future trends for all countries globally. Int. J. Electr. Power Energy Syst..

[bib67] Sarkodie S.A., Adams S., Leirvik T. (2020). Foreign direct investment and renewable energy in climate change mitigation: does governance matter?. J. Clean. Prod..

[bib68] Shin Y., Yu B., Greenwood-Nimmo M. (2011). Modelling Asymmetric Cointegration and Dynamic Multipliers in a Nonlinear ARDL Framework.

[bib69] Shokoya N.O., Raji A.K. (2019). Electricity theft: a reason to deploy smart grid in South Africa. 2019 International Conference on the Domestic Use of Energy (DUE).

[bib70] Spalding-Fecher R., Senatla M., Yamba F., Lukwesa B., Himunzowa G., Heaps C., Nyambe I. (2017). Electricity supply and demand scenarios for the Southern African power pool. Energy Pol..

[bib72] Straub S., Vellutini C., Warlters M. (2008). Infrastructure and Economic Growth in Eas Asia. (Policy Research Working Paper No. 4589, East Asia & Pacific Sustainable Development Department Operations and Policy Division).

[bib73] Surana K., Jordaan S.M. (2019). The climate mitigation opportunity behind global power transmission and distribution. Nat. Clim. Change.

[bib74] Trotter P.A. (2019). Ambitions versus policy design: addressing issues of the Power Africa initiative's quantitative targets. Energy Pol..

[bib77] World Bank (2015). World Development Indicators 2015.

[bib79] World Bank (2019). World Development Indicators.

[bib80] World Energy Outlook (2018). International Energy Association.

[bib81] Yeboua K. (2019). Foreign direct investment, financial development and economic growth in Africa: evidence from threshold modeling. Transnational Corporations Rev..

[bib82] Zheng W., Walsh P.P. (2019). Economic growth, urbanization and energy consumption—a provincial level analysis of China. Energy Econ..

